# Clinical Features and Long-Term Outcomes After Laparoscopic Surgery in Patients Co-existing With Endometriosis and Adenomyosis

**DOI:** 10.3389/fmed.2021.696374

**Published:** 2021-07-22

**Authors:** Ting-Ting Sun, Xiao-Yan Li, Jing-Hua Shi, Yu-Shi Wu, Zhi-Yue Gu, Jin-Hua Leng

**Affiliations:** Department of Obstetrics and Gynecology, Peking Union Medical College Hospital, Peking Union Medical College and Chinese Academy of Medical Science, Beijing, China

**Keywords:** endometriosis, adenomyosis, pregnancy rate, laparoscopic cystectomy, postoperative outcomes

## Abstract

**Objective:** To investigate the difference of clinical features and outcomes between EM patients with and without AM after following up for at least 6 years after surgery.

**Methods:** We retrospectively analyzed 358 EM patients who had a minimum of 6 years follow-up after laparoscopic cystectomy, which was performed by one single doctor at Peking Union Medical College Hospital from January 2009 to April 2013. All women were divided into AM group and non-AM group and analysis was performed in preoperative characteristics, surgical findings and postoperative outcomes during follow-up.

**Results:** A total of 358 EM patients were recruited, of which 142 (39.7%) were in the AM group and the rest 216 (60.3%) in the non-AM group. Between the two group, the mean age was 34.6 vs. 32.2 years (*P* < 0.001). The mean operating time in the AM and non-AM group was 73.2 vs. 61.9 min (*P* < 0.001). According to the revised AFS classification, the mean score of the two group were 60.3 vs. 45.5 (*P* < 0.001). At the end of the follow-up, though the AM group was with higher rate of disease relapse, yet no significant difference was found between the two groups in statistical comparison (34/142 [23.9%] vs. 34/216 [15.7%], *P* = 0.053). With a minimum follow-up of 6 years after laparoscopic cystectomy, failed and successful pregnancy were seen in 107/142(75.4%) and 35/142 (24.6%) patients in the AM group vs. 114/216(52.8%) and 102/216 (47.2%) patients in the non-AM group (*P* < 0.05). As for the successfully pregnant patients, live births, including spontaneous pregnancy and IVF-ET, were seen in 34/35 (97.1) vs. 99/102 (97.1) patients between AM and non-AM groups, while others ended in spontaneous abortion. No significant associations were found between the two groups in infertility, leiomyoma presence, the size of ovarian endometrioma, type of deep infiltrating endometriosis (DIE) or type of recurrence (*P* > 0.05).

**Conclusion:** Compared with non-AM group, EM patients with concurrent AM may have higher age, longer mean operating time and higher mean AFS score. In terms of fertility outcomes, patients in the AM group were with lower likelihood of pregnancy after surgery during the long-time follow-up.

## Introduction

Endometriosis (EM) is a benign gynecological disease characterized by the presence of endometrial-like tissue outside the uterine cavity (1). Meanwhile, adenomyosis (AM) refers to the invasion and growth of ectopic endometrial glands and stroma within the myometrium (2). EM occurs in 10–15% women of childbearing age and nearly half of them are concurrent with AM (3, 4). EM and AM share many common symptoms, such as chronic pelvic pain (CPP) and infertility, accounting for more than 80% of women with pelvic pain and more than 40% with decreased fecundity (5–9). Incidence of EM is gradually increasing year by year in prevalence across the population and the rate of EM coexisting with AM also increases with age, which is seriously affecting women's life quality and fertility. Despite the pathological findings, the etiology of EM and AM remains unclear, with no satisfactory clinical classification and limited treatments. For EM, endometriomas are very common type of EM occurring in 17–44% of women with EM, and laparoscopic cystectomy is known as the gold standard in diagnosis and the recommended first-line treatment (10–12). For AM, most previous studies have assessed populations of women who underwent hysterectomy to report the prevalence of AM. Up to the present, there is still a lack of high-quality data evaluating specifically EM patients concurrent with AM as for their clinical features and long-term postoperative outcomes. Data more than 5 years follow-up after surgery were not available. In this study, AM was diagnosed according to the ultrasonography criteria due to the accuracy of ultrasound (13, 14). We evaluated the prevalence of AM in patients undergoing laparoscopic surgery for EM and investigated the clinical features and postoperative outcomes between EM patients with and without AM with a minimum follow-up of 6 years. We hope to provide gynecologists with a more comprehensive understanding and better management of EM and AM.

## Methods

This is a retrospective study conducted at Department of Obstetrics and Gynecology, Peking Union Medical College Hospital (PUMCH). We identified all the patients who underwent laparoscopic cystectomy and pathologically diagnosed with EM from January 2009 to April 2013. All the surgeries were performed by one gynecologist who is expert on EM in PUMCH. This study was approved by the Ethics Committee of Peking Union Medical College Hospital (JS-1722). Written informed consent was obtained from all participants.

Inclusion criteria consist of following conditions: (1) surgically treated in the Department of Obstetrics and Gynecology of PUMCH; (2) histopathologically diagnosed with EM after operation; (3) complete clinical and pathological data; (4) premenopausal. Patients were excluded if they had previously undergone surgical treatments in other hospitals, they were pregnant, they were with malignant gynecological disease, and they received hormonal therapy (oral contraceptives, OCs; gonadotropin-releasing hormone analog, GnRHa; levonorgestrel intrauterine system) in the preceding 3 months before surgery.

The standard surgery was performed as follows: (1) A thorough inspection was made to identify the extent of lesions and pelvic adhesions. (2) All visible peritoneal implants were removed or coagulated. (3) Normal anatomy was restored as much as possible. (4) Ovarian cysts were stripped carefully to avoid unnecessary damage to the healthy tissue. As for surgical details, the presence, localization, and extent of typical powder burn and subtle lesions, adhesions, and deep infiltrating implants were recorded. Patients were scored and staged according to the revised American Fertility Society (rAFS) classification system (15). Deeply infiltrating endometriosis (DIE) was diagnosed histologically if the lesion infiltration depth was 5 mm or more under the peritoneal surface.

According to the above criteria, a total of 347 patients were diagnosed with EM after surgery. Within the whole study group, most cases with AM either underwent biopsy or didn't receive surgical treatments when the uterus was evenly enlarged. Some patients had myomectomy during surgery but postoperative pathological reports proved to be adenomyoma. Therefore, AM cases in this study was diagnosed based on pathological reports as well as imaging methods, such as ultrasound. Diagnosis of AM was made when 3 or more of the following sonographic features were present: heterogeneous myometrial echotexture (presence of an indistinctly myometrial area with decreased or increased echogenicity), globular-appearing uterus (regular enlarged uterus), asymmetrical thickness of anteroposterior wall of the myometrium, subendometrial myometrial cysts (round anechoic areas of 1–7 mm diameter), subendometrial echogenic linear striations (radiate pattern of thin acoustic shadowing not arising from echogenic foci), or poor definition of the endometrial-myometrial junction, according to previous studies (13, 14, 16–18).

The size of the ovarian endometrioma was defined as the largest diameter of cysts. The elevated serum level of CA125 is defined as >35 U/ml according to the clinical laboratory at PUMCH. The diagnosis of infertility was made when the patient had a normal sex life without using any contraception but was unable to conceive for over 1 year, after ruling out male factors of infertility. The severity of pain symptoms were defined according to the visual analog scoring (VAS) system. As for patients, mild dysmenorrhea (VAS score 1-3) means it does not affect their daily life or work and they don't need painkillers, while moderate dysmenorrhea (VAS score 4-7) on the other hand, may have effects and they will occasionally need analgesics or other treatments. Severe dysmenorrhea (VAS score 8 and above), however, will seriously affect patients' daily life, causing severe pain and symptoms like nausea, vomiting or syncope, in which case the patients must take some treatments for the pain or they will be unable to maintain normal life during every menstrual period.

Clinical features of eligible patients were retrospectively collected and analyzed after searching and reviewing medical records. The following medical information were recorded: age; body mass index (BMI); parity; surgical history; symptoms of dysmenorrhea, dyspareunia, dyschezia, dysuria, and abnormal uterine bleeding; size of endometrioma; location of cysts; serum CA125 levels; operative time; intraoperative blood loss; AFS stage. For post-operative treatments, patients with AM and with no need for reproduction received treatment of Mirena (levonorgestrel intrauterine system). Meanwhile, those with fertility needs were all treated with long-term hormone therapy. For those without AM and those without fertility requirements, OCs maintenance treatment after 3–6 injections of GnRHa were recommended. It should be noted that some patients may take the medicine intermittently and irregularly. Patients with fertility requirements may not receive very long-term hormone therapy because their desire to have children may also change. Sometimes the desire to have children was only a temporary idea.

In our center, all patients are followed up according to an internal protocol. A standard gynecological examination and a transvaginal ultrasound are conducted before surgery, at 3, 6, and 12 months after surgery, and then yearly after surgery. Menstrual-reproductive factors and symptoms of pain are also evaluated. During follow-up, patients were asked whether they experienced dysmenorrhea, pelvic pain or dyspareunia. Pain recurrence was defined as the recurrence of pain after a period of at least 3 months of relief after surgery. Endometrioma recurrence was determined using ultrasonography and defined as the presence of a persistent ovarian cyst that had a thin wall (with a diameter of at least 2 cm), regular margins, a homogenous low echogenic fluid content with scattered internal echoes and did not resolve after several successive menstrual cycles. Recurrence was identified when pain, endometrioma, or both were noted at follow-up. Postoperative information was collected in the long-term follow-up and included postoperative medications, pregnancy, related symptoms, imaging results and recurrence time.

All patients were divided into AM group and non-AM group. Analysis was performed between the two groups to compare the preoperative characteristics, surgical findings and postoperative outcomes during follow-up. IBM SPSS 23.0 software was used for statistical analysis. Continuous variables were analyzed using Mann-Whitney U test. Categorical variables were analyzed using *t*-test or Fisher's exact test. Logistic regression models were used for multivariate analysis, in which the variables included were those found to be statistically significant in the univariate analysis. All statistical tests were two-sided and differences were considered statistically significant at *P* < 0.05.

## Results

After the exclusion of 79 (18%) patients who were lost to follow-up, a total of 358 EM patients were recruited, of which 142 (39.7%) were in the AM group and the rest 216 (60.3%) in the non-AM group. The comparisons of demographic characteristics and clinical features between the two groups are showed in Table 1.

**Table 1:** Comparisons of demographic characteristics and clinical features between the AM group and non-AM group.

**Characteristics/group**	**AM**	**Non-AM**	***P***
Number (%)	142 (39.7)	216 (60.3)	
Mean Age, y	34.6 ± 5.2	32.2 ± 5.4	<0.001
Gravidity	1.37 ± 1.51	0.86 ± 1.13	0.001
Parity	0.53 ± 0.59	0.27 ± 0.47	<0.001
VAS Score	5.6 ± 3.3	4.5 ± 3.4	0.002
Time of dysmenorrhea, m	74.57 ± 84.71	52.83 ± 71.06	0.017
Degree of dysmenorrhea (%)			0.009
No	24 (16.9)	61 (28.2)	
Mild	19 (13.4)	31 (14.4)	
Moderate	27 (19.0)	51 (23.6)	
Severe	72 (50.7)	73 (33.8)	
CPP (%)	30 (21.1)	29 (13.5)	0.057
Ca125, U/ml	123.6 ± 285.92	76.14 ± 85.15	0.064
Elevated CA-125 (%)	106 (80.9)	134 (67.7)	0.008
Dyspareunia (%)	44 (31.0)	40 (18.6)	0.007
Tenesmus (%)	44 (31.0)	46 (21.4)	0.041
Infertility (%)			0.573
No	118 (83.1)	174 (80.6)	
Primary	14 (9.9)	29 (13.4)	
Secondary	10 (7.0)	13 (6.0)	
Size, cm			
Left	5.33 ± 2.1	5.45 ± 2.1	0.663
Right	5.19 ± 1.77	5.4 ± 1.98	0.392
DIE	92 (64.8)	97 (44.9)	0.433
USL	57 (62.0)	68 (70.1)	
Cul-de-sac	11 (12.0)	7 (7.2)	
Recto-vaginal Septum	4 (4.3)	6 (6.2)	
USL+ Cul-de-sac	16 (17.4)	10 (10.3)	
Ureter	4 (4.3)	6 (6.2)	
EM in douglas pouch (%)	20 (29.9)	16 (14.7)	0.015
Closed douglas pouch (%)	129 (90.8)	135 (62.5)	<0.001
Leiomyoma (%)	27 (19.3)	37 (17.2)	0.619
Operating time, min	73.22 ± 19.93	61.87 ± 22.69	<0.001
Mean bleeding volume, ml	63.59 ± 63.51	50.07 ± 79.15	0.092
rAFS score	60.26 ± 23.13	45.51 ± 25.33	<0.001
Stage (%)			0.004
I	0 (0.0)	1 (0.5)	
II	3 (2.1)	7 (3.2)	
III	39 (27.5)	95 (44.0)	
IV	100 (70.4)	113 (52.3)	
Follow-up time, m	82 [60–120]	84 [60–117]	0.199
Recurrence (%)	34 (23.9)	34 (15.7)	0.053
Type of recurrence (%)			0.189
No	108 (76.1)	182 (84.3)	
Pain	16 (11.3)	18 (8.3)	
Endometrioma	11 (7.7)	12 (5.6)	
Both	7 (4.9)	4 (1.9)	
Second surgery (%)	16 (11.3)	9 (4.2)	0.01
Pregnancy after surgery (%)			<0.05
Failed	107 (75.4)	114 (52.8)	
Pregnant	35 (24.6)	102 (47.2)	

*EM, endometriosis; AM, adenomyosis; y, year; m, month; min, minute; cm, centimeter; ml, milliliter; VAS, visual analog score; CA-125, cancer antigen 125; CPP, chronic pelvic pain; DIE, deep infiltrating endometriosis; USL, uterosacral ligament; rAFS, revised American Fertility Society*.

Between AM group and non-AM group, the mean age was 34.6 vs. 32.2 years (*P* < 0.001), the mean gravidity was 1.37 ± 1.51 vs. 0.86 ± 1.13 (*P* = 0.001), and the mean parity was 0.53 ± 0.59 vs. 0.27 ± 0.47 (*P* < 0.001). As for symptoms, CPP was present in 30 (21.1%) vs. 29 (13.5%) cases in AM group and non-AM group (*P* = 0.057). For degree of dysmenorrhea, the mean VAS score was 5.6 vs. 4.5 (*P* = 0.002). Tenesmus during menstruation were presented in 44 (31.0%) vs. 46 (21.4%) patients (*P* = 0.041). Dyspareunia was seen in 44 (31.0%) vs. 40 (18.6%) cases between the two groups (*P* = 0.007). The mean level of CA-125 were 123.6 vs. 76.1 U/ml (*P* = 0.064) while elevated CA-125 levels were found in 106 (80.9%) vs. 134 (67.7%) patients (*P* = 0.008). Before surgery, infertility was present in 24 (16.9%) vs. 42 (19.4%) cases in AM group and non-AM group. For the percentage of infertile patients, whether primary or secondary, there was no significant difference between the two groups (*P* = 0.573).

As for the surgical procedure, the mean operating time in the AM and non-AM group was 73.2 vs. 61.9 min (*P* < 0.001). There was no significant difference seen in the laterality of ovarian cysts, mean size of cysts or mean bleeding volume during operations between the two groups (*P* > 0.05). During laparoscopy, 20 (29.9%) vs. 16 (14.7%) cases were diagnosed with deep infiltrating endometriosis (DIE) lesions in Douglas pouch (*P* = 0.015). According to the revised AFS classification, the mean score of the two group were 60.3 vs. 45.5 (*P* < 0.001).

For the whole study group, the median follow-up time was 83 months, and no significant difference in the follow-up time was seen between AM and non-AM group (83.0 vs. 85.0 months, *P* = 0.199). At the end of the follow-up, though the AM group was with higher rate of disease relapse, yet no significant difference was found between the two groups in statistical comparison (34/142 [23.9%] vs. 34/216 [15.7%], *P* = 0.053). With a minimum follow-up of 6 years after laparoscopic cystectomy, failed and successful pregnancy were seen in 107/142 (75.4%) and 35/142 (24.6%) patients in the AM group vs. 114/216 (52.8%) and 102/216 (47.2%) patients in the non-AM group (*P* < 0.05). As for the successfully pregnant patients, live births, including spontaneous pregnancy and IVF-ET, were seen in 34/35 (97.1) vs. 99/102 (97.1) patients between AM and non-AM groups, while others ended in spontaneous abortion. As pregnancy results were affected by many factors, all the patients were divided into two groups according to the postoperative pregnancy outcomes. In multivariate analysis, only age and concurrent with adenomyosis were seen related to poor postoperative pregnancy outcomes (*P* < 0.05). In multivariate analysis (Table 2), however, age, CPP and adenomyosis were the independent risk factors of pregnancy outcomes between the two groups (*P* < 0.05). As for comparisons of other clinical characteristics between AM and non-AM group, no significant associations were found in infertility, leiomyoma presence, the size of ovarian endometrioma, the presence of DIE or type of recurrence (*P* > 0.05).

**Table 2:** The risk factors of postoperative pregnancy in univariate and multivariate analysis.

**Characteristics/pregnant**	**Yes**	**No**	**Univariate analysis**	**Multivariate analysis**
			***P*** **95% CI**	***P*** **95% CI**
Number	137	221		
Age [range], y	29.8 ± 3.6 [26–39]	35.2 ± 5.4 [29–43]	<0.001 0.749–0.842	<0.001 0.304–0.513
Dysmenorrhea	101/137 (73.7%)	172/221 (77.8%)		
CPP	12/137 (8.8%)	47/221 (21.3%)		0.029 0.210–0.921
DIE	65/137 (47.4%)	124/221 (56.1%)		
Adenomyosis	35/137 (25.5%)	107/221 (48.4%)	0.04 0.325–0.973	0.034 0.312–0.954
Stage III-IV	132/137 (96.4)	215/221 (97.3%)		
Recurrence	24/137 (17.5%)	44/221 (19.9%)		

*y, year; CPP, chronic pelvic pain; DIE, deep infiltrating endometriosis; CI, confidence interval*.

## Discussion

Both AM and EM are with the characteristics of estrogen dependence and more commonly seen in women of reproductive age. Ovarian endometriomas are very common type of EM and laparoscopic surgery has been the recommended gold standard for women with endometriomas in both diagnosis and treatment (11, 12). In this study, we investigated the clinical features of patients with concurrent endometriosis and adenomyosis and all patients were followed up regularly for 6–10 years. We found that women with both EM and AM may have a lower pregnancy rate.

EM and AM are both characterized by the presence of ectopic endometrial glands and stroma outside the uterine cavity, except that AM lesions are within the myometrium and EM lesions are locating in the pelvic. Therefore, many studies have reported that EM and AM share not only some common clinical symptoms but also possibly common pathogenesis. They suggested that endometrial stroma being in direct contact with the underlying myometrium allows communication and interaction, thus facilitating endometrial invagination or invasion of a structurally weakened myometrium during periods of regeneration, healing, and reepithelization (19–22). Till now, the etiology of EM and AM is still not completely understood yet and more molecular studies are required to elucidate their pathogenic mechanisms.

Over the past decade, the rate of EM concurrent with AM has been demonstrated to increase with age, which is seriously affecting women's life quality and fertility. In this study, we found that 39.7% of the patients with EM were with AM, which is in accordance with previous studies reporting that 34.6–79% of patients with EM could be combined with AM (4, 9, 18, 23). They also reported that AM becomes more common in the later reproductive years. According to our results, the mean age of patients in the AM group were slightly higher than the non-AM group, which was in an agreement with previous studies showing a linkage between increasing age and risk of AM (4, 18).

Clinically, diagnosing AM based on its symptoms, such as chronic pelvic pain and dysmenorrhea, is not practical because of the similarity with other gynecologic pathologies and lack of specificity, which makes the preoperative diagnosis extremely difficult (24, 25). There have been studies that included mainly women with severe symptoms reporting the incidence of AM as high as 50% (25, 26). These patients had a higher possibility of AM than the general population, thus the results in these studies might have been overestimated. On the other hand, pathological diagnosis is made based on the ectopic endometrium including stroma and glands. In the past, a diagnosis of AM is often established only at histological examination of uterus, limiting the rate of AM to patients who received hysterectomy. According to pathological reports, the incidence of AM ranges from 5 to 70%, depending on the histological criteria and the number of sections examined (2, 27, 28). Due to the risks of surgery in AM patients, it is difficult to completely remove the lesions for conservative surgery. After surgery, the integrity of myometrium wall may be damaged and adhesion of uterus may get worse, thus increasing the difficulty of postoperative pregnancy. Therefore, pathological diagnosis of AM is limited in clinical practice, which makes imaging evidence the most important non-surgical diagnostic method.

Ultrasound is the most widely used non-invasive diagnostic method with convenient application and low price. According to previous studies, its sensitivity and specificity in the diagnosis of AM were 72–82% and 81–85%, respectively (17). Magnetic resonance imaging (MRI) is also a widely recognized imaging method for the diagnosis of AM, with similarly high diagnostic accuracy. It can provide multidimensional images, help clinicians understand the distribution of lesions comprehensively, specify disease classifications and guide the selection of treatments. Its sensitivity and specificity have been reported to be 77% and 89%, respectively (29). A review concluded that transvaginal ultrasound (TVS) should be the primary tool for the diagnosis of AM, with MRI being combined when TVS is inconclusive (13). Our diagnosis of AM was made based on pathological reports as well as imaging methods, which were mostly by ultrasound and a small part by MRI. Nowadays, TVS and MRI have shown high accuracy in the diagnosis of AM and been commonly used in preoperative diagnosis. According to previous reports, the diagnostic accuracy based on imaging evidence in EM concurrent with AM is about 80% (16, 18, 30). What is more, in this study, the diagnosis of AM was made by the combination of pathological reports and imaging evidence, which means the results are reliable and accurate.

According to our results, patients in the AM group were associated with higher parity. A review in 2013 suggested that one of the main risk factors of AM was having had more than one pregnancy (31). Shrestha et al. found that the rate of AM was higher in parous women than in the nulliparous (32). In agreement with our data, many other studies have reported that AM was related to advanced age and multiparity (33, 34).

As for clinical symptoms, EM and AM have much similarity, such as CPP and infertility, which are the most commonly recognized reasons for women with pelvic pain and decreased fecundity (4). Previous studies have reported that AM is an independently risk factor for pelvic EM and constitutes an important reason for sterility in patients with EM (23, 30). This is consistent with our results that EM patients have lower pregnancy rate after surgery when they were concomitant with AM.

CPP is well-known to be an adding factor related to AM in women with EM. It can make further explanation to our results that a larger proportion of patients in the AM group were with CPP and higher VAS score of dysmenorrhea. Moreover, a study reported that 43% of patients with EM were identified to be coexisting with AM when they had severe dysmenorrhea, deep dyspareunia and EM of the rectosigmoid (35). These results were in accordance with ours that dyspareunia and tenesmus during menstruation were more presented in patients of the AM group.

According to previous reports, EM and AM can have synergistic effects on the fertility of women and cause a variety of pregnancy complications. Conservative surgery is one of the main surgical treatment methods for patients who have fertility requirements when they were concurrent with AM and EM (36). During the surgical procedure, our results showed that the mean operating time in the AM group was significantly longer than that of the non-AM group. Similarly, there have been some studies reporting that the operation time was longer and intraoperative bleeding was more in patients with AM compared with EM alone (37). Uteruses in AM cases have unclear boundaries of lesions and they were prone to have recurrence after surgery, with poor healing ability of myometrium. As consequences of relating surgery in the uterus diagnosed with AM, these patients are associated with perinatal complications such as abortion, premature delivery, placenta previa, placental implantation, postpartum hemorrhage and uterine rupture (38).

In our study, we also found that patients in the AM group were with higher rate of disease relapse in the long-term follow-up, though there was no statistical difference between the two groups. In accordance with our results, previous studies reported that patients with AM were usually related to deep EM and more advanced stage of EM, leading to poor prognosis (39). Our results also showed higher AFS score in the AM group. With a minimum follow-up of 6 years after surgery, the AM group were with higher proportion of failed pregnancy and lower rate of pregnancy, regardless of spontaneous or IVF-ET pregnancy. The possible reason is that AM is significantly associated with patients' infertility, which had been reported by many studies (23, 31, 40). In our previous study, we investigated the fertility outcomes after laparoscopic cystectomy in infertile patients. Our results showed age, CPP and AM were the independent risk factors of pregnancy outcomes for patients with infertility (41). Combined with the results of this study, we could see that no matter whether the patients were infertile before surgery, they may have lower pregnancy rate when they were with higher age, CPP and AM.

As far as we are aware, the major strength of our study is that compared with previous studies, our data were collected after a real long-term follow-up, which was 6–10 years. For our patients, all the surgeries were performed primarily by one surgeon, which can eliminate many inherent confounding factors like introducing selection bias. As a retrospective study, the main limitations of our study were small sample size and recall bias. In the future, more prospective studies of patients coexisting with EM and AM are required to establish a predictive model for clinicians to make a better understanding of the diagnosis, management and prognosis.

## Conclusion

This study summarized the clinical features, surgical finds and postoperative results of patients concurrent with EM and AM in a long follow-up. Compared with non-AM group, EM patients coexisting with AM were significantly correlated with higher age, longer mean operating time and higher mean AFS score. Significant differences were also found between two groups in terms of fertility outcomes as patients in the AM group have lower pregnancy rate during the long-time follow-up. Therefore, gynecologists should be aware of the clinical characteristics of EM patients when they are concurrent with AM, for these patients may have higher rate of recurrence and lower rate of pregnancy after surgery. Further prospective studies are required to develop a model to help with the diagnosis, management and prognosis of EM and AM.

## Data Availability Statement

The original contributions presented in the study are included in the article/supplementary material, further inquiries can be directed to the corresponding author/s.

## Author Contributions

J-HL developed the idea for the project. The study was designed by T-TS and X-YL. J-HS, Y-SW, and Z-YG performed the data analysis and takes full responsibility for the integrity of the data. T-TS and X-YL drafted the manuscript with inputs and critical discussion from J-HL. All authors approved the final version.

## Conflict of Interest

The authors declare that the research was conducted in the absence of any commercial or financial relationships that could be construed as a potential conflict of interest.
